# *Neisseria subflava* Type 6 Secretion System competition with bacterial and fungal species

**DOI:** 10.1007/s00253-026-13870-6

**Published:** 2026-05-23

**Authors:** Alan Calder, Lori A. S. Snyder

**Affiliations:** School of Life Sciences, Pharmacy, and Chemistry, Kingston University, Penrhyn Road, Kingston upon Thames, KT1 2EE UK

**Keywords:** Type 6 Secretion System, Type VI Secretion System, T6SS, Toxin antitoxin system, *Neisseria* spp., Commensal *Neisseria*

## Abstract

**Abstract:**

The aim of this study was to assess whether a Type VI Secretion System (T6SS) present in the commensal, non-pathogenic *Neisseria subflava* strain KU1003-01 plays a role in competition between these bacteria and other commensal and pathogenic Gram-negative microorganisms and the yeast *Candida albicans*. Using an overlap extension PCR strategy, the core T6SS gene encoding membrane protein TssM was knocked out for *N. subflava* strain KU1003-01. Co-culture competition assays were conducted with either the *N. subflava* or *N. subflava tssM* knock-out strains as the attackers and *N. gonorrhoeae* strain NCCP11945, *N. subflava* strain KU1003-02, or *C. albicans* strain 3153 as the targets. There was a significant difference in recoverable target cell CFUs observed following co-culture with the T6SS knock-out strain in comparison to the parent strain. TssM has been identified as critical to T6SS function for a number of species, and our findings indicate that the T6SS in *N. subflava* contributes to competition with these other species.

**Key points:**

• *Neisseria subflava Type 6 Secretion System is functional*

• *Neisseria subflava attacks other microbes of the same niche*

• *Neisseria subflava is competent for transformation*

**Supplementary information:**

The online version contains supplementary material available at 10.1007/s00253-026-13870-6.

## Introduction

Type VI Secretion Systems (T6SS) are complex multi-protein systems that are estimated to be present in at least one quarter of all Gram-negative bacterial species (Bingle et al. 2008; Boyer et al. 2009), including *Neisseria subflava* and other non-pathogenic *Neisseria* spp. (Calder and Snyder 2023). Although it has been speculated that the T6SS may have evolved as a means to increase competitive fitness (Cianfanelli et al. 2016), T6SS can contribute to virulence for some pathogenic species (Hopf et al. 2014; Ma et al. 2014; Clemens et al. 2018). Other potential roles of the T6SS include host cell adhesion (Lertpiriyapong et al. 2012; Liu et al. 2015; Liaw et al. 2019), nutrient acquisition (Wang et al. 2015; Lin et al. 2017; Si et al. 2017; Li et al. 2022), motility (Montenegro et al. 2021), and biofilm formation (Kim et al. 2017). T6SSs are well known for contact-dependent toxin secretion; however, more recent studies have shown that T6SS effectors can also be secreted through mechanisms that are independent of cell-to-cell contact (Song et al. 2021).

It was once thought that T6SS could only act against Gram-negative species, due to the thick cell walls of Gram-positive bacterial species being considered unbreachable by the T6SS spike. It is now known that T6SS can act against Gram-positive bacteria (Le et al. 2021; Pei et al. 2022) as well as fungal species (Trunk et al. 2018, 2019; Luo et al. 2023). In regard to anti-fungal T6SS, *Pseudomonas syringae* was the first species reported to have a T6SS that acted against yeast cells (Haapalainen et al. 2012).

T6SSs are usually composed of 13 core proteins: TssA; TssB; TssC; TssD (Hcp); TssE; TssF; TssG; TssH (ClpV); TssI (VgrG); TssJ; TssK; TssL; and TssM (Bingle et al. 2008; Coulthurst 2013; Morgado and Vicente 2022). The core proteins are divided into three groups based on the secretion system subunit they co-assemble. TssJLM forms the membrane complex, TssAEFGK forms the baseplate complex, and TssBC forms the needle sheath of the injection apparatus (Yang et al. 2018). When fully assembled, T6SS forms peptidoglycan-anchored trans-envelope channels (Santin et al. 2019) that have similarities to inverted bacteriophage tails (Coulthurst 2013).

In a functioning T6SS, contraction of the TssBC sheath drives a nanotube structure formed of TssD (Hcp) “rings” (Ballister et al. 2008) topped with a TssI, a valine-glycine repeat (VgrG) “spike” protein (Alcoforado et al. 2015) into target cells. VgrG facilitates the delivery of effector proteins into target cells (Cianfanelli et al. 2016), and following delivery, the contracted TssBC sheaths are disassembled by the T6SS AAA + ATPase, TssH (ClpV) (Bönemann et al. 2009; Zoued et al. 2014).

The ability of the T6SS to kill competitor target cells has been demonstrated through mutation or knock-out of key T6SS genes; mutants have been shown to have reduced virulence or an inability to kill competitors (Custodio et al. 2021; Stietz et al. 2020; Wang et al. 2021). The effects of *tssM* mutants on T6SS function have been documented for a range of species that include *Acidovorax citrulli* (Fei et al. 2022; Pei et al. 2022), *A. baumannii* (Repizo et al. 2015; Li et al. 2019; Le et al. 2021), *Burkholderia cenocepacia* (Aubert et al. 2015), *Campylobacter jejuni* (Bleumink-Pluym et al. 2013), *E. coli* (de Pace et al. 2011), *Pseudomonas aeruginosa* (Lin et al. 2015), *Ralstonia solanacearum* (Zhang et al. 2012; Asolkar and Ramesh 2020), *Vibrio cholerae* (Stietz et al. 2020), and *Neisseria cinerea* (Custodio et al. 2021). For some species, *tssM* mutants have been used experimentally as T6SS-deficient controls (Fei et al. 2022).

TssM is a key structural component of the T6SS involved in forming the trans-envelope membrane complex (MC) (Felisberto-Rodrigues et al. 2011; Zoued et al. 2016) through associations with the inner membrane lipoprotein TssL and the outer membrane lipoprotein TssJ (Durand et al. 2015; Cianfanelli et al. 2016). Once formed, the MC docks with the T6SS baseplate complex (BC); the cytoplasmic domains of TssM and TssL, located at the base of the membrane complex, interact with the TssG/TssK and TssK/TssE baseplate subunits (Logger et al. 2016; Zoued et al. 2018; Liebl et al. 2019). In addition to TssM being required for production of the T6SS MC, this protein may have other roles including the formation of transient outer membrane pores (Durand et al. 2015) and providing ATPase activity required for assembly of the TssM-TssL-Hcp Complex (Ma et al. 2012).

When functioning as a contact-dependent secretion system, the T6SS “injects” toxins into neighbouring cells (Ben-Yaakov and Salomon 2019). In regard to *Neisseria* spp., a number of the commensal and pathogenic *Neisseria* spp. are specific to human hosts and therefore have the potential to interact with one another (Higashi et al. 2011; Baerentsen et al. 2022). Although *Neisseria* spp. were once considered to have their own niche environments, this being the oro-nasopharynx for the commensals and *N. meningitidis* (Johnson 1983; Uwamino et al. 2017; Weyand, 2017; Diallo et al. 2019; Dorey et al. 2019) and the genital tract for *N. gonorrhoeae* (Spinosa et al. 2007), *N. subflava* has been found to colonise genitourinary areas (Baraldes et al. 2000) as well as the gastrointestinal tract (Niikura et al. 2023). Similarly, over the past few decades, *N. gonorrhoeae* and *N. meningitidis* have been isolated from other host sites including the oropharynx, rectum and eye (Morris et al. 2006; Mitchell et al. 2008; Bissessor et al. 2015: Tao et al. 2016; Ladhani et al. 2020; Tsakalos et al. 2021; Butler et al. 2022).

In regard to *C. albicans*, like *Neisseria* spp., this can also colonise the oral cavity, genital areas, and gastrointestinal tract including the rectum (Kumamoto 2011; Shirvani et al. 2021). *Neisseria* spp. and *C. albicans* are both occasionally isolated from the same clinical sample and these species most likely exist together at specific anatomical sites. While this has been shown to be the case for *C. albicans* and *N. gonorrhoea*e (Hipp et al. 1975), this is also likely to be the case for *N. subflava* and *C. albicans.* Unlike Candida*, Neisseria* spp. are considered to be primary colonisers within the oral cavity (Sedghi et al. 2021) and Candida may require the presence of established, primary colonisers in order to adhere (Sultan et al. 2018).

Social interactions between bacterial species within a niche are a common phenomenon (Copeland et al. 2024) and horizontal gene transfer (HGT) between species is a form of social cooperation (Lee et al. 2022). T6SSs are known to have a diverse range of effectors (Smith et al. 2025) and genes encoding these components have been identified on mobile as well as integrative conjugative elements (Coyne et al. 2016; García-Bayona et al. 2021). Auxiliary T6SS gene clusters are thought to be horizontally shared (Jana et al. 2022), and although diversification of T6SS toxin arrays may help prevent the evolution of resistance in competitors (Smith et al. 2025), diversification can also lead to social incompatibilities within a niche. It has been shown that social compatibility between parent strains and offspring can be abolished upon introduction of unique T6SS toxins (Vassallo et al. 2020).

In this study, we generate a *tssM* knock-out strain of *N. subflava* strain KU1003-01 and demonstrate that the T6SS plays a role in competition against both commensal and pathogenic bacterial species as well as yeast. Although the antibacterial activity of T6SS was first described over a decade ago, to our knowledge, this is the first report documenting the role of the commensal *N. subflava* T6SS in competition with other bacterial and fungal species.

## Methods

### Strains and growth conditions

The cells used for this study were grown from a freezer stock of the original isolate. *Neisseria subflava* strain KU1003-01 and *N. subflava* strain KU1003-02 were first isolated in the Spring of 2012 following throat sampling of 64 student volunteers on three separate occasions (Calder et al. 2020). *Neisseria gonorrhoeae* strain NCCP11945 was received from the National Institute of Health, Korea Centres for Disease Control and Prevention (Chung et al. 2008). *C. albicans* strain 3153 was donated by Dr. Suzy Moody of Kingston University. All bacterial and fungal species were grown at 37 °C with 5% CO2 on GC agar (Oxoid) supplemented with Kellogg’s (Kellogg et al. 1963) and 5% Fe (NO3)_3_. For selection of T6SS knock-out mutants, the GC agar was supplemented with 100 µg/ml kanamycin (Sigma-Aldrich). To select for surviving target cells following competition, the growth medium was supplemented with 2 µg/ml ciprofloxacin (Sigma-Aldrich). *N. subflava* KU1003-01 is sensitive to ciprofloxacin, whilst the target cells were all resistant at this concentration.

### Construction of *N. subflava* T6SS mutant

A splice overlap extension PCR (SOE PCR) (Horton et al. 1989; Wörmann et al. 2016) was used to create a *tssM* knock-out strain. The oligonucleotide primers were ordered from Sigma-Aldrich (Supplementary Table 1). Genomic DNA was extracted from the *N. subflava* strain KU1003-01 using a Gentra Puregene Kit (Qiagen). Using a Q5 High-Fidelity 2X Master Mix kit (New England Biolabs), primer pairs HA1 Fwd with HA1 Rev and HA2 Fwd with HA2 Rev were then used to amplify a 196 bp region (HA1) upstream and a 213 bp region downstream (HA2) of *tssM*. A third 851 bp fragment representing the coding sequence for the kanamycin resistance gene (*kan*) was amplified using primer pair Kan-Fwd and Kan-Rev using the 1221 bp EZ-Tn5™ < KAN-2 > Transposon (Lucigen) as a template. The HA1, HA2, and Kan PCR products were added to splice overlap extension (SOE) PCR as follows: HA1 and Kan were added along with primer pair HA1-Fwd and Kan-Rev to generate a 1014 bp fragment (HA1 + Kan). The HA1 + Kan and HA2 PCR products were then used as templates along with the primer pairs HA1-Fwd and HA2-Rev to yield the final 1188 bp SOE construct, HA1 + Kan + HA2. At all stages, PCR products were first sized on a 2% agarose E-Gel (Invitrogen), and bands of the expected size were excised and cleaned up using a Wizard® SV Gel and PCR Clean-Up System (Promega).

### Transformation of *N. subflava* strain KU1003-01

The final PCR construct was introduced into *N. subflava* strain KU1003-01 using a variation of the method of Gunn and Stein (1996) as follows: 10 µl suspensions of piliated *N. subflava* strain KU1003-01 were spotted onto GC agar (Oxoid) supplemented with Kellogg’s supplement (Kellogg et al. 1963), 5% Fe(NO3)_3_, and magnesium chloride (Sigma-Aldrich) at a final concentration of 2mM. The spots were allowed to dry before the PCR construct was added at around 1 µg DNA per spot. The plates were incubated right side up at 37 °C with 5% CO_2_ for 5 h, after which, growth was scraped from the spots using a sterile loop and passed onto fresh GC agar containing kanamycin at 100 µg/ml.

### Knockout confirmation using PCR

Following transformation, all kanamycin-resistant colonies of *N. subflava* strain KU1003-01 were first passed onto fresh GC agar (Oxoid) supplemented with Kellogg’s (Kellogg et al. 1963), 5% Fe(NO3)_3_, and 100 µg/ml kanamycin and incubated overnight at 37 °C in 5% CO2. The presence of kanamycin cassettes in the resistant isolates was first confirmed by PCR using primers HA1-Fwd and HA2-Rev following genomic DNA extraction using a Gentra Puregene Kit (Qiagen). Sizing of PCR products was carried out using the precast 2% E-Gel electrophoresis system (Invitrogen).

### *N. subflava* strain KU1003-01 parent and knock-out strain sequencing

A chosen isolate identified as producing a PCR product of around 1188 bp, indicative of *tssM* knock-out, and the parent strain were used for genomic DNA extraction using the Gentra Puregene Kit (Qiagen). The parent and mutant genomic DNA were sent to MicrobesNG (Birmingham, UK) for Illumina whole genome sequencing and de novo read assembly. After assembly of the sequence data by MicrobesNG, the parent strain assembled into 70 contigs (https://www.ncbi.nlm.nih.gov/nuccore/JBQLII000000000) and the *tssM* mutant strain into 71 contigs (https://www.ncbi.nlm.nih.gov/nuccore/JBQLIJ000000000).

### Bacterial and fungal competition assays

The competition assay was adapted from Murdoch et al. (2011) and is detailed as follows. Cells were taken from overnight growth on GC agar and suspended in sterile 1 × phosphate-buffered saline (PBS) (Sigma-Aldrich). The cell suspensions were then adjusted to an OD of 0.5 at 600 nm. A 1:1 (attacker: target) ratio was used for all competition assays, with the attackers being either the *N. subflava* KU1003-01 parent or *tssM* knock-out strains and the targets being *N. gonorrhoeae* strain NCCP11945, *N. subflava* strain KU1003-02, or *C. albicans* strain 3153. Using a 5-mm-diameter cork borer, rings were aseptically marked on the surface of the GC agar competition plates. Into each ring, 10 µl volumes of target cells were first spotted onto GC agar (Oxoid) supplemented with Kellogg’s (Kellogg et al. 1963), 5% Fe(NO3)_3_, and the spots were allowed to dry at 37 °C for 15 min before the addition of either parent or *tssM* knock-out strains of *N. subflava* KU1003-01. The co-culture spots were grown on GC agar at 37 °C in 5% CO_2_ for 24 h after which the spots were aseptically scraped from the plates and suspended in sterile 1 × PBS (Sigma-Aldrich). Surviving colony-forming units (CFUs) of target cells were determined by serial dilution and growth on GC media containing ciprofloxacin at 2 µg/ml.

### Calculating recovered CFU/mL for target cells following competition

These were estimated using the formula CFU/mL = (number of colonies × DF)/*V*, where DF represents the dilution factor and* V* represents the volume inoculated in mL.

### Statistical analysis

Unless stated, experiments were performed in triplicate, and the data were analysed using SPSS (version 28) with *P*-values of < 0.05 being considered significant. The competition data is presented as the arithmetic mean of replicates ± SD.

### RNA-seq

RNA was extracted from the *N. subflava* strain KU1003-01 and the *tssM* mutant using the RNAprotect Bacteria Reagent kit (QIAGEN) using Protocols 6 and 7. RNA was stored at −80 °C. RNA integrity was determined using the Agilent 2100 Bioanalyzer with RNA 6000 Nano kit to produce an RNA Integrity Number (RIN). RNA was quantified using Qubit 2.0 Fluorometer with Qubit RNA Broad Range assay kits (Molecular Probes) according to the manufacturer’s instructions. RNA purity was assessed on a BioDrop Duo spectrophotometer at 230, 260, and 280 nm. RNA-seq was conducted by Novogene. Reads were visualised against the genome sequence using Integrative Genomics Viewer (IGV; Robinson et al. 2011).

## Results

### Generation of *N. subflava* strain KU1003-01 lacking tssM

**Fig. 1:**
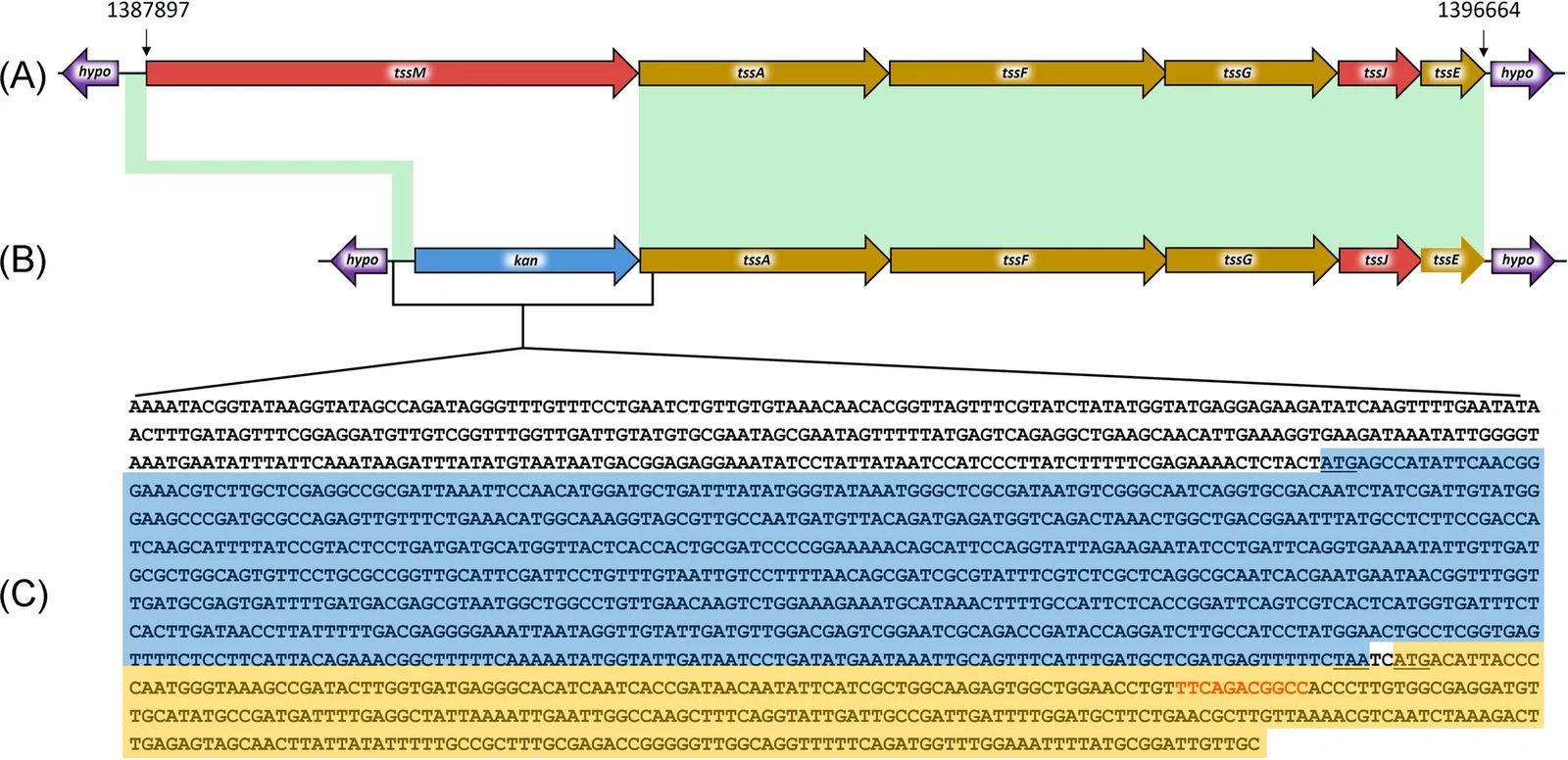
**A** A graphical representation of T6SS core cluster A located between positions 1,387,897 and 1,396,664 in *N. subflava* strain KU1003-01. Genes involved in membrane complex formation are shown in red, and the baseplate complex in gold. Hypothetical non-T6SS genes (hypo) are shown in purple. **B** A representation of the *tssM* knock-out showing insertion of the kanamycin resistance cassette. Regions with light-green shading joining A and B highlight sequences identified with 100% identity between the parent and *tssM* knock-out sequences. **C** A portion of the Illumina sequence data for the *tssM* knock-out strain with the sequence for the kanamycin resistance gene highlighted in blue and the first 337 nucleotides of *tssA* highlighted in gold. Start and stop codons for each of the genes are underlined and the location of the 11mer DUSvar1 sequence included in the PCR construct is shown in red text

*N. subflava* strain KU1003-01 was transformed with a PCR construct designed to knock out *tssM* of the Type 6 Secretion System and replace it with a kanamycin resistance cassette (Fig. 1). Successful recovery of kanamycin-resistant colonies after transformation with the PCR construct demonstrates transformability of the commensal *N. subflava* KU1003-01. Antibiotic resistance testing identified the *N. subflava* KU1003-01 parental strain as having a kanamycin MIC of around 25 µg/ml. Following transformation, this increased to 100 µg/ml for the transformed strain. Transformation with the PCR construct was further confirmed via PCR, generating a product of the desired size to have knocked-out *tssM* and inserted the kanamycin resistance cassette. Growth curve analysis revealed no significant differences between the parent and mutant under normal growth conditions (data not presented), as has been observed for other species (Li et al. 2019; Song et al. 2020; De Oliveira et al. 2021; Montenegro Benavides et al. 2021).

### Confirmation of the tssM knock-out using whole genome sequencing

To further confirm the generation of the mutant, Illumina sequencing was conducted on the parent and knock-out strains. According to the Illumina sequencing data for the knock-out strain (MicrobesNG), the PCR construct was integrated into the correct genomic location and successfully replaced *tssM*. To further investigate differences between the parent and knock-out genome sequences, as well as the MinION-enhanced genome sequence from *N. subflava* KU1003-01 (Calder and Snyder 2023), these were aligned using Mauve (Rissman et al. 2009), and SNP files were generated. No polymorphisms within any of the coding or non-coding regions could be identified between the parent strain, *tssM* knock-out strain, and the MinION-enhanced genome sequences, other than the intended, engineered knock-out sequence.

### RNA-seq demonstrates expression of tssM in the parent strain but not in the mutant

**Fig. 2:**
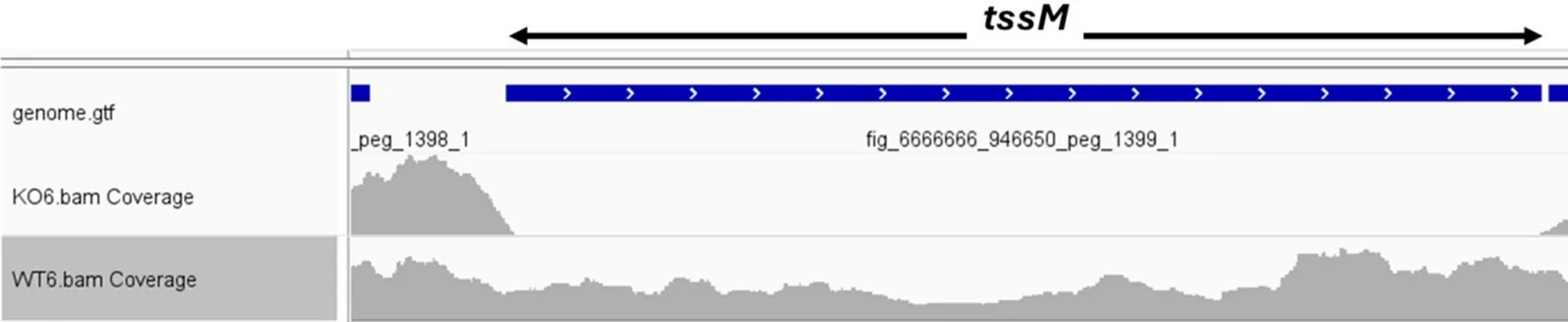
Output from the Integrative Genomics Viewer (IGV; Robinson et al. 2011) showing all RNA-seq reads mapped to the parent (WT) and *tssM* knock-out (KO) of *N. subflava* KU1003-01. Reads are shown in grey, here illustrating that there is no expression of *tssM* in the knock-out mutant strain

Extracted RNA from the *N. subflava* strain KU1003-1 parent strain had a RIN of 9.5 and a concentration of 79 ng/µl. The *tssM* mutant RNA had a RIN of 7.1 and a concentration of 111 ng/µl. These are within the Novogene parameters of ≥ 6.0 and ≥ 50 ng/µl. RNA-seq QC from Novogene indicated 2.1 Gbp of clean bases in the parent and 3.6 Gbp in the mutant. The Q20 scores were 98.62% for the parent and 98.81% for the mutant. There were a total of 14,104,422 reads in the parent strain, of which 13,753,003 mapped to the *N. subflava* KU1003-01 reference genome sequence, a mapping rate of 97.51%. In the mutant, there were 23,710,014 reads in total and 23,298,097 mapped to the genome sequence, a rate of 98.26%. Gene expression analysis showed that the *tssM* gene is expressed in the parent strain, but not in the mutant (Fig. 2; Table 1). Table 1 shows RNA-seq expression data for the core T6SS cluster.

**Table 1:** RNA-seq data for the core T6SS cluster in *N. subflava* KU1003-01 parent (WT) and *tssM* mutant (KO)

Gene				Read counts		FPKM^1^		KO vs WT		
Name	Start	End	Strand	WT	KO	WT	KO	log_2_ FC^2^	*P*-value	padj
*vgrG1*	1018014	1020497	−	299	1512	40.7032	95.1925	1.24	0.004	0.040
*clpV*	1020642	1023455	−	23	84	2.7638	4.6683	0.77	0.175	0.428
*hcp*	1023527	1024015	−	6	3	4.1491	0.9594	−2.04	0.144	0.378
*tagL*	1024086	1025714	−	4	13	0.8303	1.2480	0.59	0.653	0.846
*tssL*	1025732	1026394	−	3	12	1.5301	2.8305	0.88	0.624	0.836
*tssK*	1026396	1027811	−	6	27	1.4328	2.9820	1.06	0.171	0.419
*tssB*	1027902	1028402	+	7	19	4.7246	5.9309	0.34	0.732	0.893
*tssC*	1028441	1029976	+	38	195	8.3657	19.8539	1.26	0.010	0.076
*paar*	1030029	1030292	−	264	665	338.1492	393.9310	0.23	0.586	0.816
*tssM*	1387897	1391172	+	3628	294	374.4827	14.0348	−4.72	1.77E−22	4.35E−19
*tssA*	1391175	1392797	+	1579	4156	328.9819	400.4603	0.30	0.473	0.734
*tssF*	1392807	1394573	+	2059	4160	394.0290	368.1792	−0.08	0.838	0.946
*tssG*	1394537	1395646	+	1053	3225	320.7848	454.3695	0.52	0.214	0.475
*tssJ*	1395666	1396205	+	1072	1915	671.2889	554.5970	−0.26	0.528	0.773
*tssE*	1396445	1396618	+	370	511	719.0530	459.2764	−0.63	0.136	0.367

^1^*FPKM*, fragments per kilobase of transcript sequence per million base pair sequenced. Normalised read counts

^2^*log*_*2*_* FC*, log_2_ fold change

Data provided by Novogene

TssM is a known key structural protein that is critical to T6SS assembly and function (Felisberto-Rodrigues et al. 2011). Even when other core T6SS genes are expressed, without TssM, the secretion system will not form (Custodio et al. 2021). Numerous other researchers have shown that without TssM, Hcp secretion is diminished (Bleumink-Pluym et al. 2013) and the TssBC sheath does not assemble (Liebl et al. 2019; Stietz et al. 2020). TssM knock-outs have been shown to be less able to kill prey species (Pei et al. 2022; Fei et al. 2022; Repizo et al. 2015; Li et al*.* 2019; Aubert et al. 2015; Bleumink-Pluym et al. 2013; de Pace et al. 2011; Lin et al. 2015; Zhang et al. 2012; Asolkar and Ramesh, 2020; Stietz et al. 2020; Custodio et al. 2021).

### Demonstration of the function of the Type 6 Secretion System in competition against other microbes

**Fig. 3:**
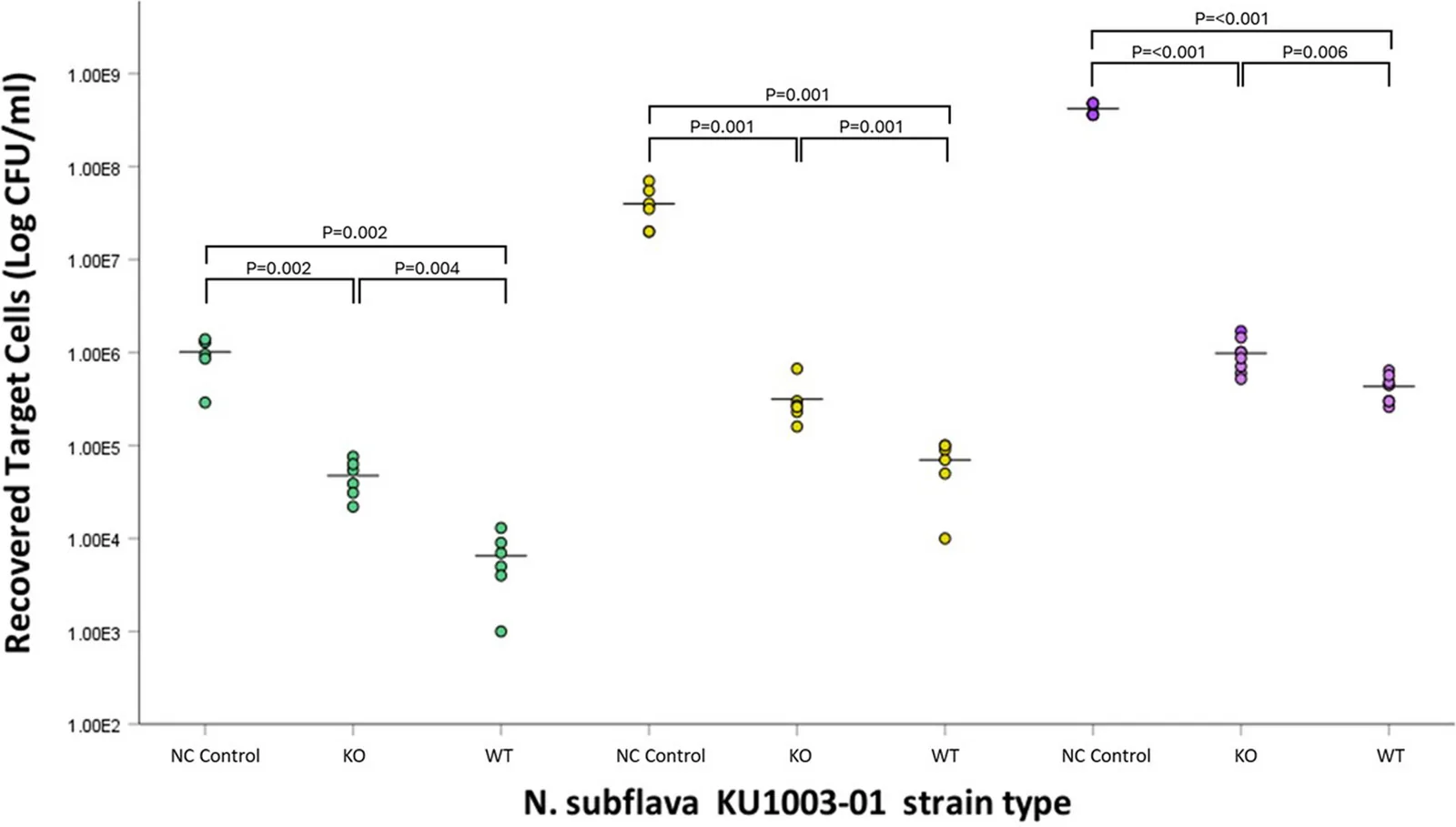
Surviving CFU/ml for *N. gonorrhoeae* strain NCCP11945 (Green), *N. subflava* strain KU1003-02 (Yellow), and *C. albicans* strain *3153* (Purple) following competition with either the *N. subflava* KU1003-01 parent (WT) or *tssM* knock-out (KO) attacker strains. Assays were performed in triplicate on three separate occasions (*n* = 9) with each assay triplicate including no-competition controls (NC) containing only target cells. Mean values are represented by bars within each data point cluster. Data from the experimental replicates were analysed using an unpaired two-tailed Student’s *t*-test for pairwise comparison with the calculated *P*-values shown on the graph

The parent and knock-out strains were used as attacker strains in competition assays versus the target bacterial pathogen *N. gonorrhoeae*, another strain of *N. subflava* that was isolated from the same host niche (KU1003-02), and the fungal pathogen *C. albicans*. Significant differences are observable in recovered target cell CFUs following co-culture with either the *N. subflava* strain KU1003-01 parent or T6SS knock-out strains (Fig. 3). Greater killing by the parental strain with its intact T6SS demonstrates that this system plays a role in competition between *N. subflava* strain KU1003-01 and other microbes of the nasopharyngeal niche. Demonstration of competition between the knock-out attacker and the target microbes suggests there may be other systems involved as well.

## Discussion

### *N. subflava *strain KU1003-01 is competent for transformation

There are only a few published studies that have confirmed commensal *Neisseria* spp. including *Neisseria elongata*, *Neisseria mucosa*, *Neisseria sicca*, *Neisseria cinerea*, *Neisseria musculi*, and *Neisseria macaque* take up and transform exogenous DNA (Gunn and Stein 1996; Dillard 2011; Harris-Jones et al. 2024). Most *Neisseria* spp. transformation investigations carried out to date have focussed on the role of the Type IV pilus and acquisition of antimicrobial resistance factors by the pathogens from commensal *Neisseria* spp. (Bowler et al. 1994; Higashi et al. 2011; Chen et al. 2020; Manoharan-Basil et al. 2021).

For *Neisseria gonorrhoeae*, only piliated cells are naturally transformable (Dillard and Chan 2024) and functioning pili (Fussenegger et al. 1997; Wolfgang et al. 1998) as well as the presence of short, conserved DNA Uptake Sequences (DUS) of between 10 and 12 nt in exogenous DNA enhance the likelihood for its uptake and transformation (Goodman and Scocca 1988; Ambur et al. 2007; Duffin and Seifert 2010; Hepp et al. 2016). The overall dominant DUS type within the genome sequences of the pathogens is known as “classical” DUS or AT-DUS and consists of ATGCCGTCTGAA (Berry et al. 2013; Frye et al. 2013). For *Neisseria subflava* strain KU1003-01, as well as other *N. subflava* biovar *flavescens*, and *N. elongata*, the overall dominant type is known as DUSvar1 or AG-DUS that consists of AGGCCGTCTGAA. For *N. mucosa* and *N. sicca*, the dominant type is known as DUSvar2 or AG-mucDUS and consists of AGGTCGTCTGAA (Berry et al. 2013; Frye et al. 2013). Copies of DUS associated with *penA* in *N. subflava* have been used to enhance the DNA uptake of the region into *N. gonorrhoeae* (Kanesaka et al. 2022). In this case, when the *penA* gene was used without DUS in the donor DNA, no transformants were obtained, emphasizing the need for DUS.

Linear DNA cannot be used to transform most bacteria, and constructs inserted into circular plasmids are usually introduced to recipient cells using methods like electroporation (Heap et al. 2012). Unlike electroporation or transformation methods that are carried out in liquid culture, piliated *N. gonorrhoeae* are more efficient at taking up DNA from purified PCR products or linearised plasmids added to solid media at high concentrations (Dillard and Chan 2024). Under situations where DUS are lacking or where large plasmids are to be introduced, other methods of transformation can be used and protocols used for the transformation of both commensal and pathogenic *Neisseria* spp. have been documented (Dillard 2011; Dillard and Chan 2024).

All *Neisseria* spp. are considered naturally competent for transformation; however, the commensals are documented as less transformable than *N. gonorrhoeae* under standard laboratory conditions (Dillard 2011). The commensals are however thought to have similar requirements to the pathogens for DNA uptake and transformation (Berry et al. 2013). Based on available evidence that the DUS enhances DNA uptake and transformation (Goodman and Scocca 1988; Ambur et al. 2007; Duffin and Seifert 2010; Hepp et al. 2016; Berry et al. 2013; Frye et al. 2013; Kanesaka et al. 2022), the PCR construct for the *tssM* knock-out strain includes a commensal DUSvar1 (Fig. 1). Successful recovery of kanamycin-resistant colonies that generated the expected PCR product size demonstrates that *N. subflava* strain KU1003-01 is naturally competent for transformation as are other *Neisseria* spp.

### Illumina whole genome sequencing for the tssM parental and knock-out strains

Active T6SS can have fitness effects that vary depending on species (Salomon 2016; Kostiuk et al. 2021; Li et al. 2021), and studies investigating knockouts for systems other than the T6SS have shown that where gene knockouts cause alterations to overall fitness, bacteria can adapt and evolve compensatory mutations (Hottes et al., 2013; McCloskey et al. 2018; Patel and Matange 2021). While mutations that alter regulatory mechanisms can occur quickly, other mutations occur over longer timescales (Carroll and Marx 2013; Patel and Matange 2021). Few genetic complementation tools exist for *Neisseria* spp., and all were developed for *N. gonorrhoeae* (Wuckelt et al. 2024; Ramsey et al. 2012). Due to the incompatibility of complementation plasmids, a few chromosomal sites in *N. gonorrhoeae* have been used for gene complementation following knock-out (Ramsey et al. 2012); however, these sites have not yet been characterised for *N. subflava* (Davey and Valdivia 2020). It was therefore important that whole genome sequencing of the parental and knock-out strains was conducted and compared to one another and to the original *N. subflava* strain KU1003-01 complete circular genome generated with MinION and Illumina (Calder and Snyder 2023). With no changes noted between the sequences, further analyses of the parent and T6SS knock-out could be investigated.

### The T6SS of *N. subflava* strain KU1003-01 contributes to competition with other bacterial as well as fungal species

The T6SS contributes to competition between *N. subflava* strain KU1003-01 and the pathogen *N. gonorrhoeae* strain NCCP11945 as well as the commensal, *N. subflava* strain KU1003-02, and the yeast *C. albicans* strain 3153 (Fig. 3). These were specifically chosen for the competition assay due to their ability to colonise the human throat. *N. subflava* KU1003-01 was isolated from a throat swab of a healthy volunteer (Calder et al. 2020). *N. subflava* KU1003-02 came from the same sample of the same volunteer. Pathogens that may colonise the pharynx include *N. gonorrhoeae* (Takahashi et al. 2008) and *C. albicans* (Bertolini et al. 2021). Therefore, these three are ideal prey for the competition assay as they are able to colonise the same niche.

For *Neisseria* spp., positioning within mixed communities can be influenced by type IV pili, and target species or strains may segregate from their attackers, moving to the expanding edge of colonies grown on solid media to improve their chances of survival (Oldewurtel et al. 2015; Zöllner et al. 2017; Custodio et al. 2021). In regard to our competition assays, attacker and target cells were inoculated into defined areas on the competition plates at concentrations suitable to produce a “lawn growth”. The addition of target cells to the plates before the *N. subflava* KU1003-01 attacker strains was done to prevent separation of the species into microcolonies, as seen when competition spots were inoculated using lower cell concentrations; this also limited the possibility of target cells “escaping” to the outer boundaries of the competition spots. Examples of competition spots both with and without defined 5-mm boundaries as well as a competition spot consisting of an attacker *N. subflava* KU1003-01 and a target *N. gonorrhoeae* inoculated from a 1:1 mixture containing cells at low concentrations are shown in Supplementary Fig. 1.

In regard to co-culture incubation times, these vary across different studies. For example, for this study, competition spots consisting of attacker and target cells were grown for a period of 24 h. This is in contrast to harvesting competition spots after only 4 h for *N. cinerea* (Custodio et al. 2021). For reference strains of *N. gonorrhoeae*, lag phases have been shown to last up to 4 h, and a steep decline in growth is seen only after 36 h (Foerster et al. 2016). Allowing competition between the attacker and target cells to proceed over a longer time period eliminated difficulties in effectively recovering cells from the competition spots after a short period of growth. In addition, while the i3 type T6SS in *N. cinerea* is thought to be constitutively active (Custodio et al. 2021), the phase of growth where the i2 type T6SS (T6SS-A) in *N. subflava* strain KU1003-01 (Calder and Snyder 2023) is most active is not yet known.

The competition environment is artificial, and it is not likely that *N. subflava* strain KU1003-01 would interact in the same way naturally with other strains of *N. subflava*, *N. gonorrhoeae*, or *C. albicans*. As a contact-dependent system, the T6SS has a short range with activity that can become self-limiting. Generally, it is thought that during competition, dead target cells can accumulate and form an interface that can prevent further attacks (Smith et al. 2020). It is possible this may be a contributing factor to any variations seen in surviving target cell CFUs across the assays.

### Competition may be multifactorial

The data from the competition assays suggest the T6SS *N. subflava* strain KU1003-01 is involved in competition with *N. gonorrhoeae*, other *N. subflava*, and *C. albicans* (Fig. 3). The T6SS however may not be the only factor responsible for the reduction in recovered CFUs. While a significant difference exists in recovered target cell CFU following competition with either the T6SS parent or the *tssM* knock-out strains, a significant difference also exists between CFU recovered from the no-competition controls and the parent and knock-out competition spots. It is notable that T6SS investigations do not tend to report a no-competition control; this may therefore be a typical result for these systems, or it may be that there are other factors involved in *N. subflava* competition with other microorganisms.

Data from RNA-seq (Table 1) shows that some of the T6SS core genes are still transcribed (*tssAEFGJ*, *paar*, and *vgrG*1). This may account for the results (Fig. 3). The gene *tssJ* encodes a protein that is part of the membrane complex with TssM and TssL, whilst *tssAEFG* are parts of the baseplate complex with TssK. It may be that the continued expression of these genes creates a means for the effector proteins to target competitor cells, although this has not been seen in other species (de Pace et al. 2011; Zhang et al. 2012; Bleumink-Pluym et al. 2013; Aubert et al. 2015; Lin et al. 2015; Repizo et al. 2015; Li et al. 2019; Asolkar and Ramesh 2020; Stietz et al. 2020; Custodio et al. 2021; Le et al. 2021; Fei et al. 2022; Pei et al. 2022). It is also possible that the continued expression of *vgrG* genes in the core and elsewhere on the chromosome enables the partial function of effectors released from the cell during the natural process of autolysis known in this species (Lesher et al. 1977).

Competition between bacterial species is often multifactorial, and individual species are thought to use a range of mechanisms including combinations of both exploitative and interference mechanisms (Stubbendieck and Straight 2016). While exploitative competition can provide benefits through enhanced nutrient acquisition or efficient surface binding, interference competition mechanisms used by commensals usually involve the secretion of antimicrobial toxins (Abt and Pamer 2014; Khare and Tavazoie 2015).

In regard to possible alternative mechanisms used by *N. subflava* strain KU1003-01 for interference competition, these could include toxin secretion by a contact-dependent secretion system other than the T6SS or through toxin secretion into the extracellular space via a contact-independent secretion system (Hibbing et al. 2010; Blanchard et al. 2014; Bauer et al. 2018; Ramamoorthy et al. 2024; Song et al. 2024).

One example of diffusible contact-independent toxins is bacteriocins (Sharp et al. 2017; Darbandi et al. 2022), and early studies into bacteriocins produced by *Neisseria* spp. indicated that some strains of *N. gonorrhoeae* produced gonocins (Flynn and McEntegart 1972; Lawton et al. 1976) and *N. meningitidis* produced meningocins; both of these bacteriocin types were found to inhibit growth of other bacterial species (Kingsbury et al. 1966; Allunans et al. 2008). Although most bacterial species are thought to be able to produce at least one bacteriocin type (Sharp et al. 2017; Darbandi et al. 2022), it is not thought that *N. subflava* strain KU1003-01 secretes diffusible toxins for competition. Using a simple “spot on the lawn” assay (Van Reenen et al. 1998; Brown et al. 2011), *N. subflava* strain KU1003-01 did not produce zones of inhibition on a lawn of *N. gonorrhoeae* (Supplementary Fig. 1).

Genes encoding a putative Type V Secretion System (T5SS) are present in *N. subflava* strain KU1003-01; like the T6SS, these are also contact-dependent inhibition (CDI) systems (Fan et al. 2016) and known to regulate growth of neighbouring cells (Jamet et al. 2015). The inhibitory effect of CDIs can be attributed to C-terminal toxin domains of CdiA (CdiA-CT) (Ikryannikova et al. 2020), and for the *N. subflava* strain KU1003-01, a putative CdiA, Ntox50 C-terminal domain protein is encoded between positions 2,165,738 and 2,165,866. The immunity protein (CdiI) is encoded between positions 2,166,228 and 2,166,527.

CDI systems are also present in *N. gonorrhoeae* strain NCCP11945 (Jamet et al. 2015) as well as other strains of *N. meningitidis* and *N. gonorrhoeae*. For *N. meningitidis*, CDIs are involved in competition and niche adaptation (Jamet and Nassif 2015; Jamet et al. 2015). For the *Neisseria* spp. pathogens, CDIs are arranged as polymorphic *maf* loci with some strains having multiple CDI-associated toxin/immunity genes (Tan et al. 2015).

Although toxin secretion within mixed bacterial populations can be induced through competition for space and nutrients (Cornforth and Foster 2013; Li et al. 2022), it is not clear from this work whether specific signals are responsible for T6SS regulation in *N. subflava* strain KU103-01. The T6SS-A in *N. subflava* is similar to the SecReT6 T6SS subtype i2 (Calder and Snyder 2023), and i2 T6SS types in other species are predicted to be regulated by specific signals, for example pH and oxidative stress for the i2-type T6SS in *Klebsiella* spp. (Storey et al. 2020). Signals for T6SS regulation in other species also include oxidative stress (DeShazer 2019; Storey et al. 2020), alterations to pH or temperature (Zhang et al. 2023), and plasma membrane perturbations (Basler et al. 2013; Lin et al. 2019).

### Interactions between commensal *Neisseria* and other microorganisms

Both *N. subflava* and the *Neisseria* spp. pathogens readily autoaggregate (Ledder et al. 2008; Bonazzi et al. 2018); however, *N. subflava* does not co-aggregate readily with other oral bacterial species (Ledder et al. 2008). In regard to *N. subflava* within mixed biofilm communities, these are usually found as “islands” within confluent high-biomass regions while other commensal *Neisseria* spp. appear dispersed (Palmer 2017).

Currently, there are only a limited number of studies published on the interactions between commensal and pathogenic *Neisseria* spp. as well as interactions between *Neisseria* spp. and fungi. While studies have focussed on the interactions between *N. gonorrhoeae* and *Candida* spp. (Hipp et al. 1975), interactions between commensal and pathogenic *Neisseria* spp. have only been visualised between *N. elongata* subspecies *glycolytica* (ATCC29315) and *N. gonorrhoeae* strain MS11. These two species have been shown to form intimate connections through their Type 4 pili (Higashi et al. 2011).

## Conclusion

This is the first study to show that the *N. subflava* KU1003-01 T6SS previously identified through genomic analysis (Calder et al. 2020; Calder and Snyder 2023) is biologically active and involved in competition with other organisms that colonise the same niche. Future work will involve the analysis of effector protein genes and generation of knock-out mutants of these effector protein genes with the aim of finding out which effectors or combination of effectors are secreted by the T6SS of *N. subflava* KU1003-01 to target either bacterial or fungal cells. It may be possible that the neisserial T6SSs could be exploited in the future as a biotechnological means to control or prevent infections by antibiotic-resistant pathogenic *Neisseria* spp. and/or other pathogenic bacterial or fungal species.

## Supplementary information

Below is the link to the electronic supplementary material.

ESM 1 (JPG 1.65 MB)

ESM 2 (JPG 1.63 MB)

ESM 3 (JPG 1.54 MB)

ESM 4 (JPG 1.96 MB)

ESM 5 (JPG 1.69 MB)

ESM 6 (JPG 1.60 MB)

ESM 7 (JPG 1.59 MB)

ESM 8 (PDF 310 KB)

## Data Availability

All data is presented here with the exception of genome sequencing data, which is available via the accession information in the Methods.
