# Supplementary figures and images for: Neisseria subflava Type 6 Secretion System competition with bacterial and fungal species
Source: Appl Microbiol Biotechnol. 2026 May 23;110(1):219. doi: 10.1007/s00253-026-13870-6 (PMC13375697; doi:10.1007/s00253-026-13870-6)

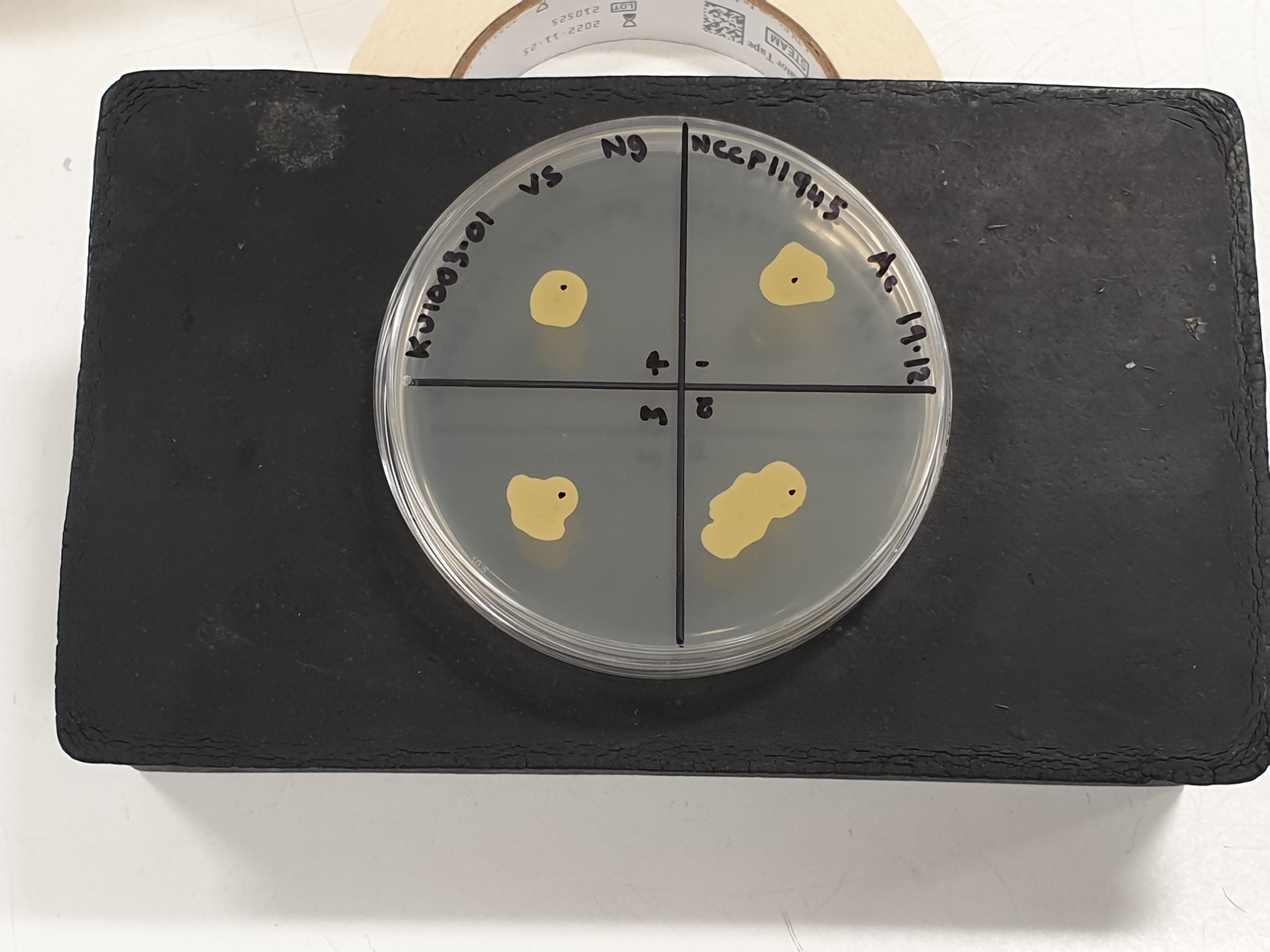

Supplement: Supplementary file 1 — (JPG 1.65 MB) [file 253_2026_13870_MOESM1_ESM.jpg]

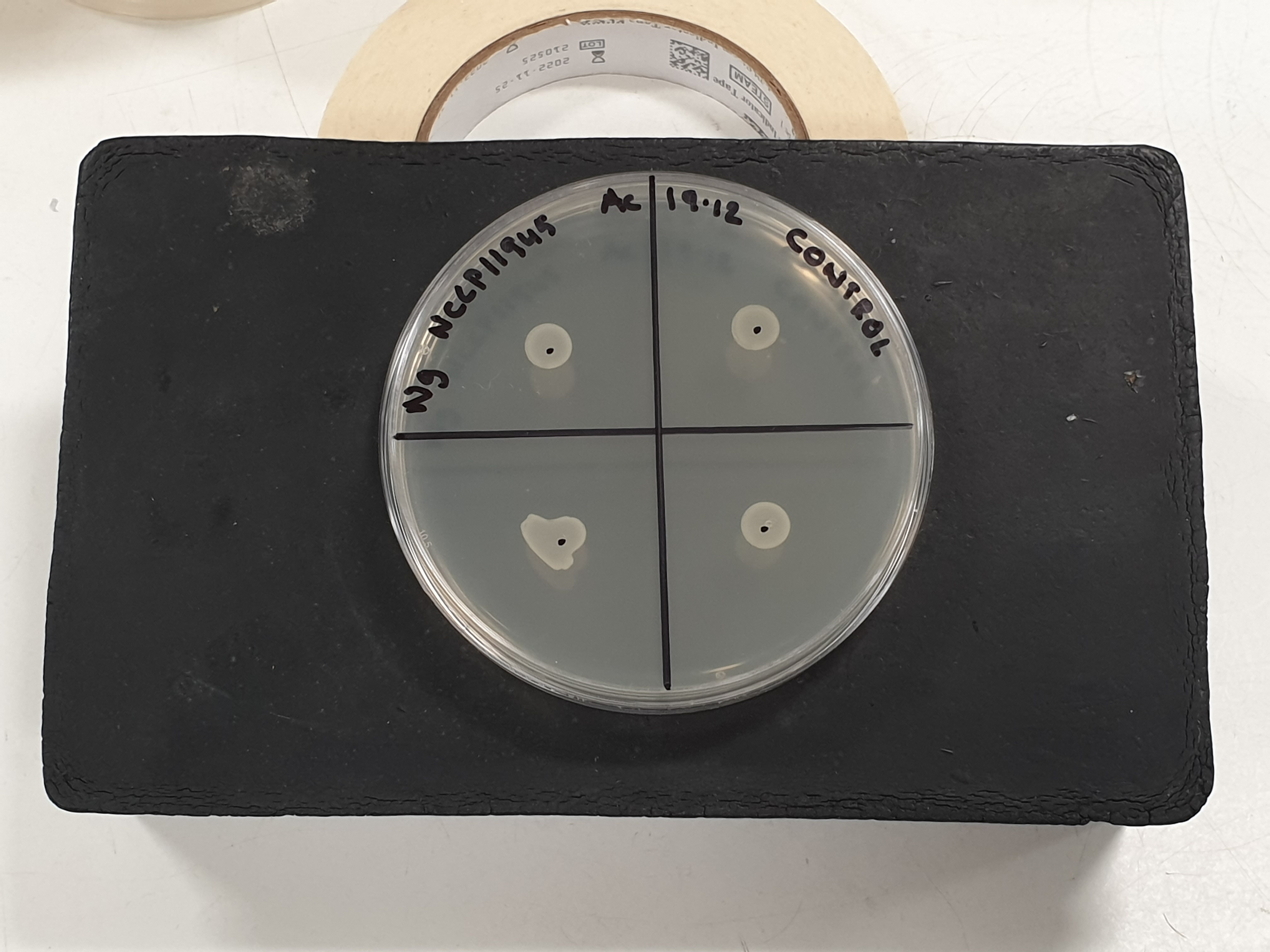

Supplement: Supplementary file 2 — (JPG 1.63 MB) [file 253_2026_13870_MOESM2_ESM.jpg]

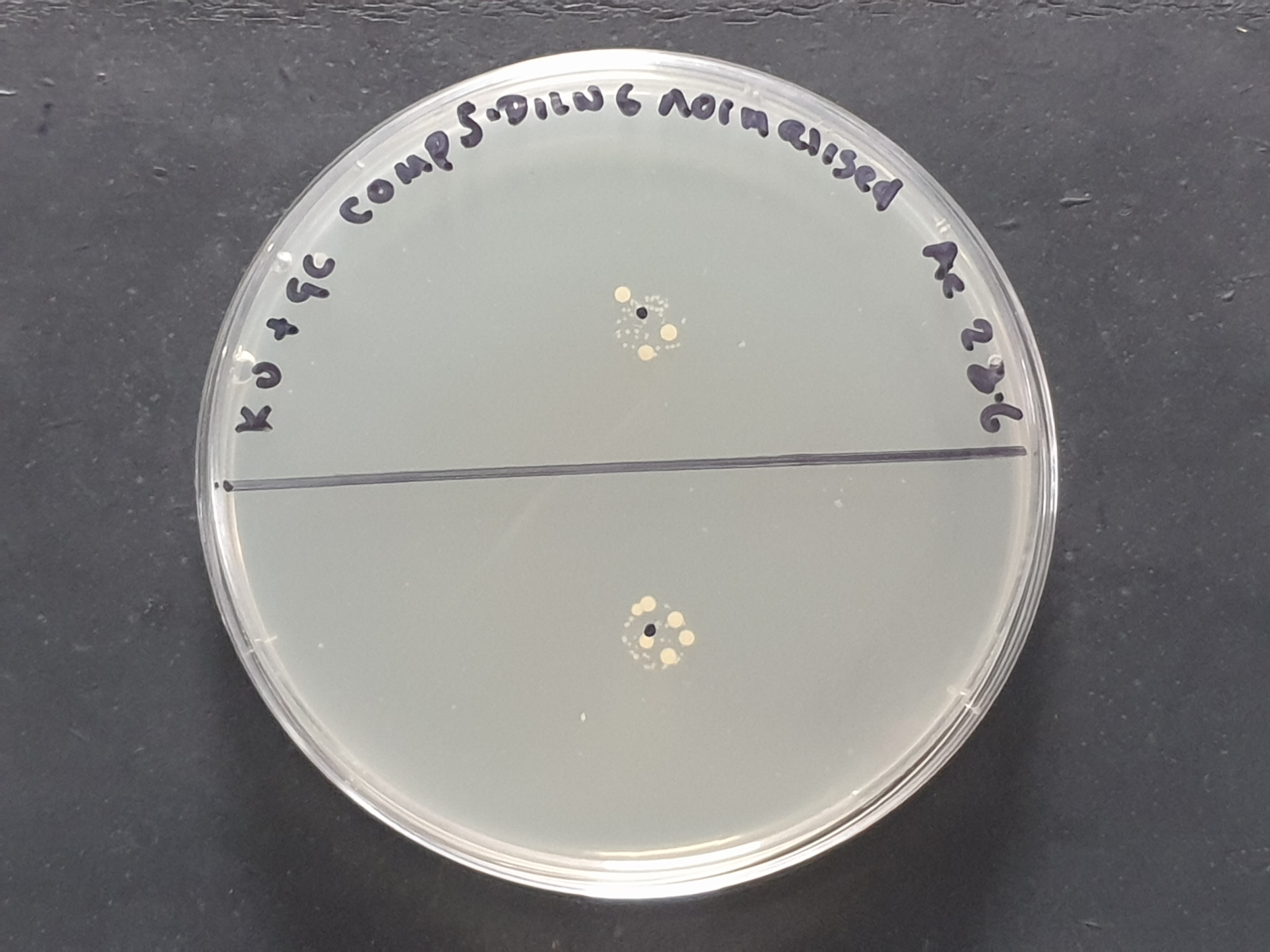

Supplement: Supplementary file 3 — (JPG 1.54 MB) [file 253_2026_13870_MOESM3_ESM.jpg]

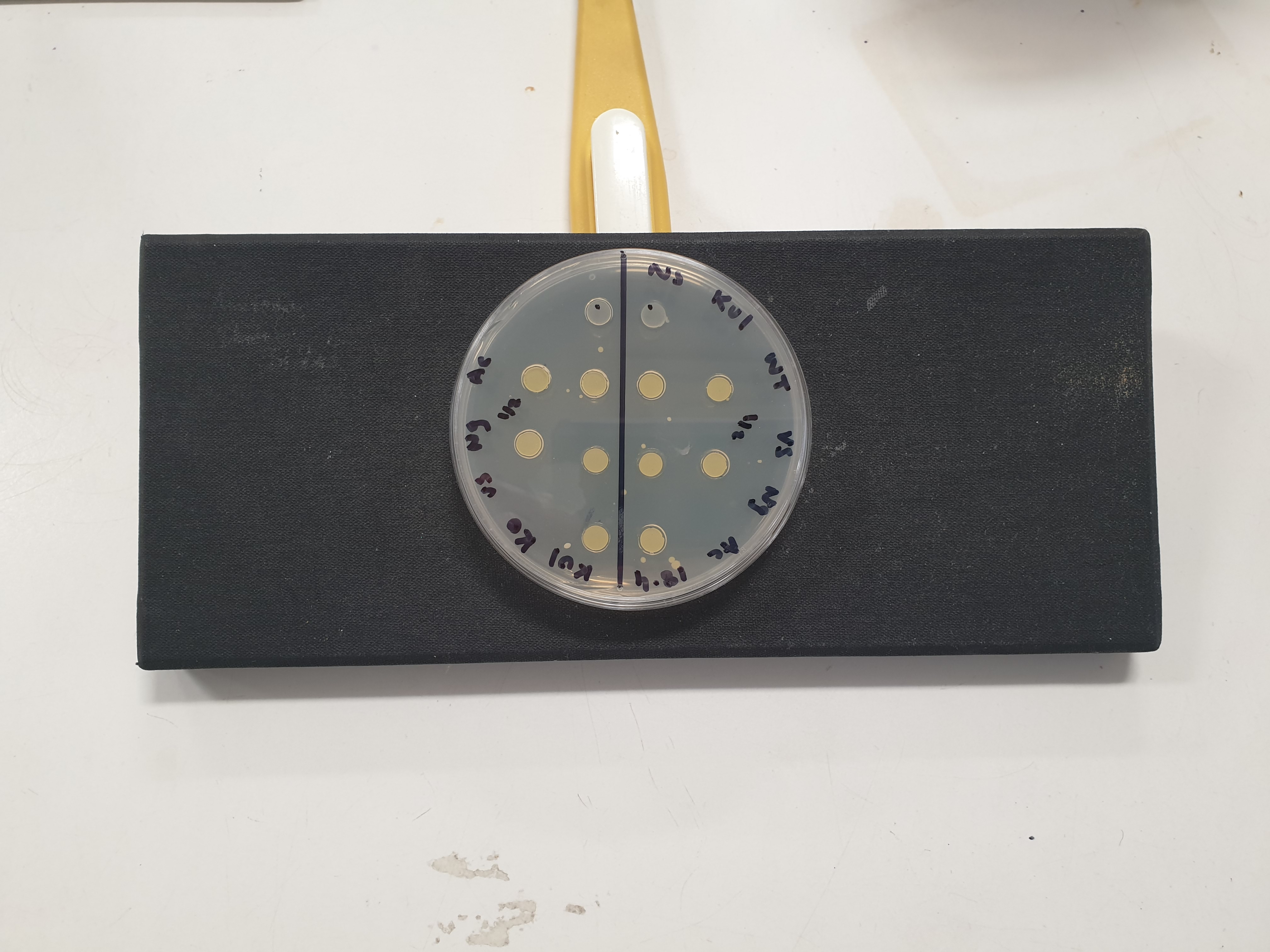

Supplement: Supplementary file 4 — (JPG 1.96 MB) [file 253_2026_13870_MOESM4_ESM.jpg]

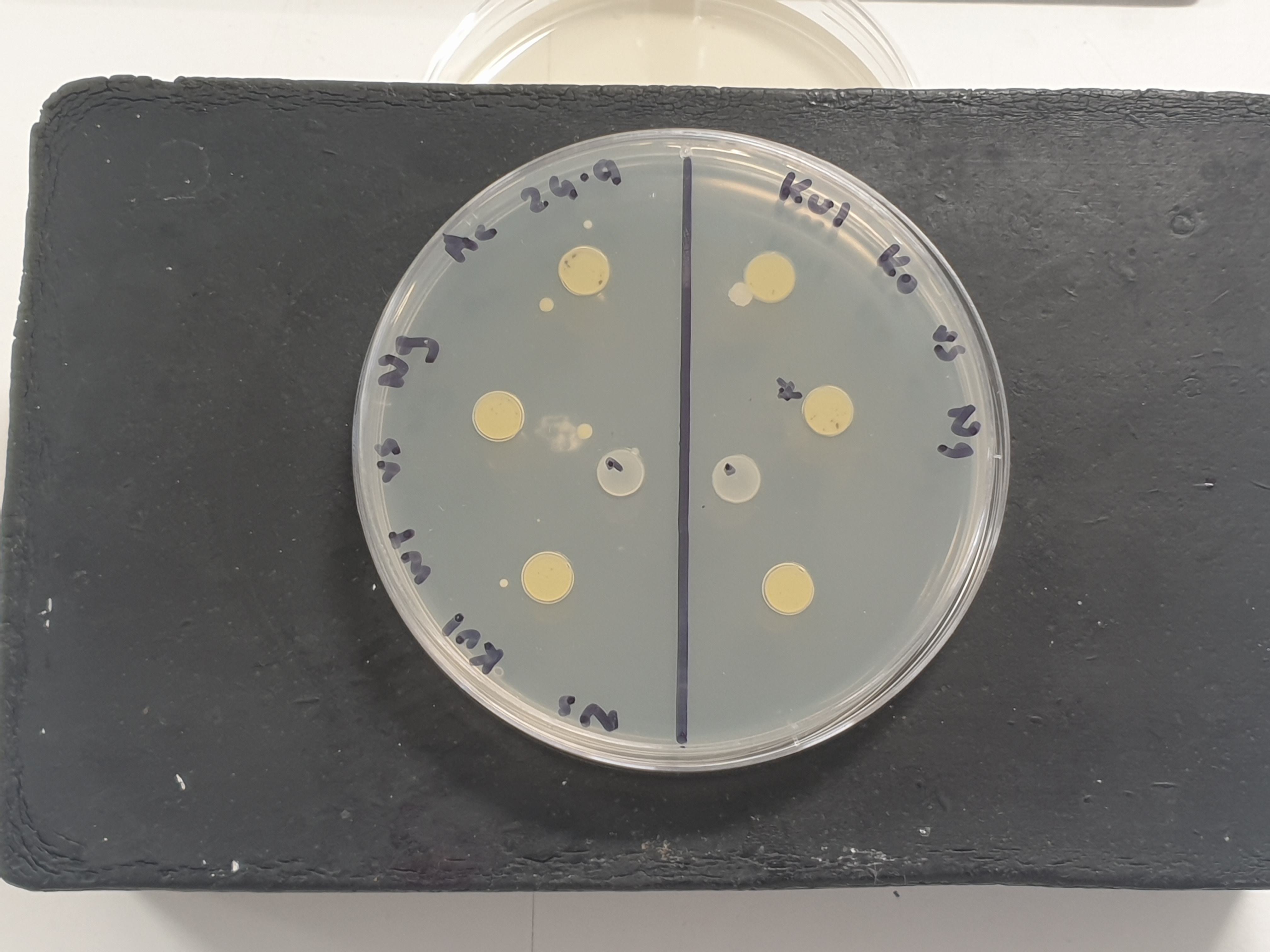

Supplement: Supplementary file 5 — (JPG 1.69 MB) [file 253_2026_13870_MOESM5_ESM.jpg]

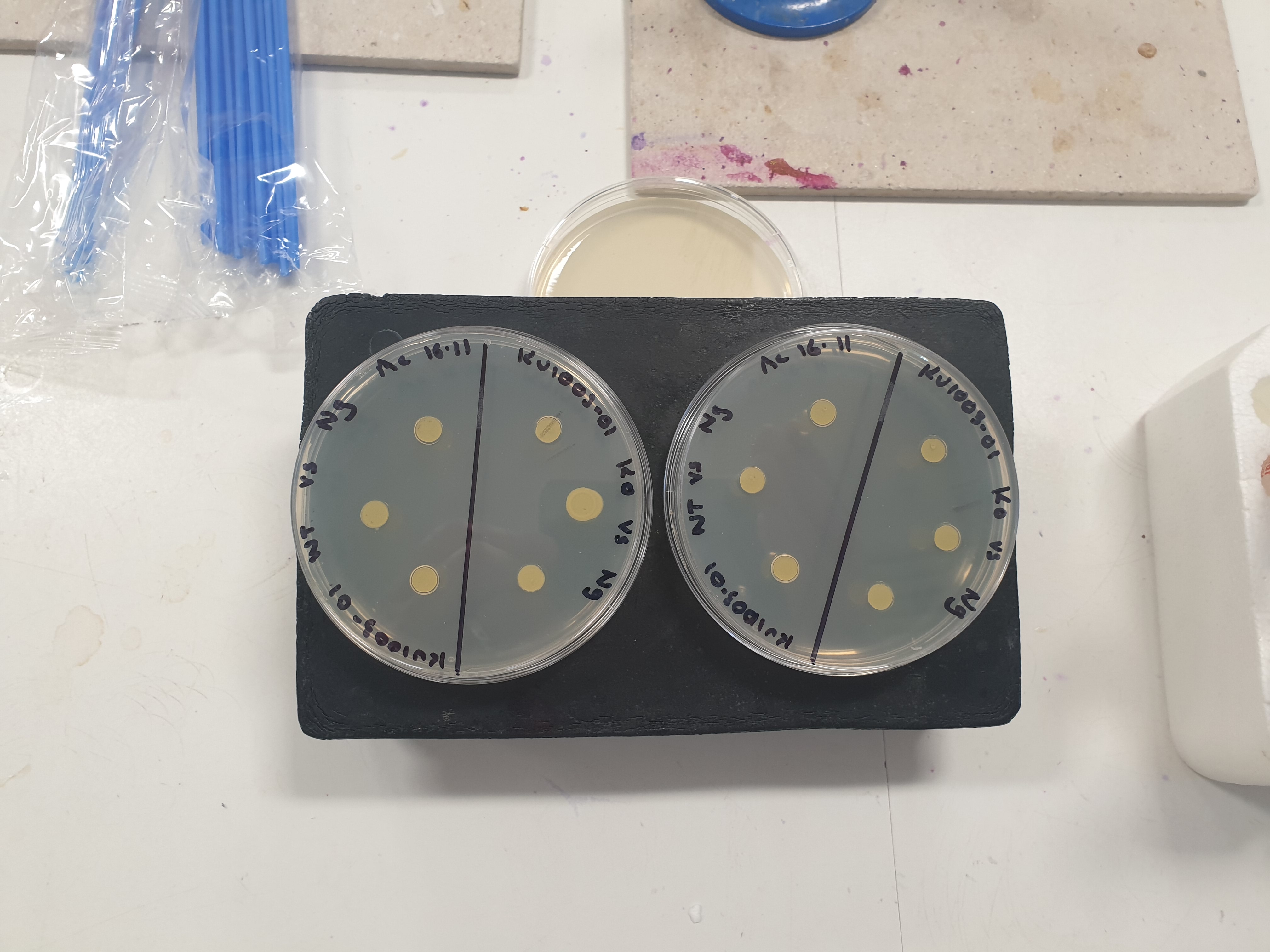

Supplement: Supplementary file 6 — (JPG 1.60 MB) [file 253_2026_13870_MOESM6_ESM.jpg]

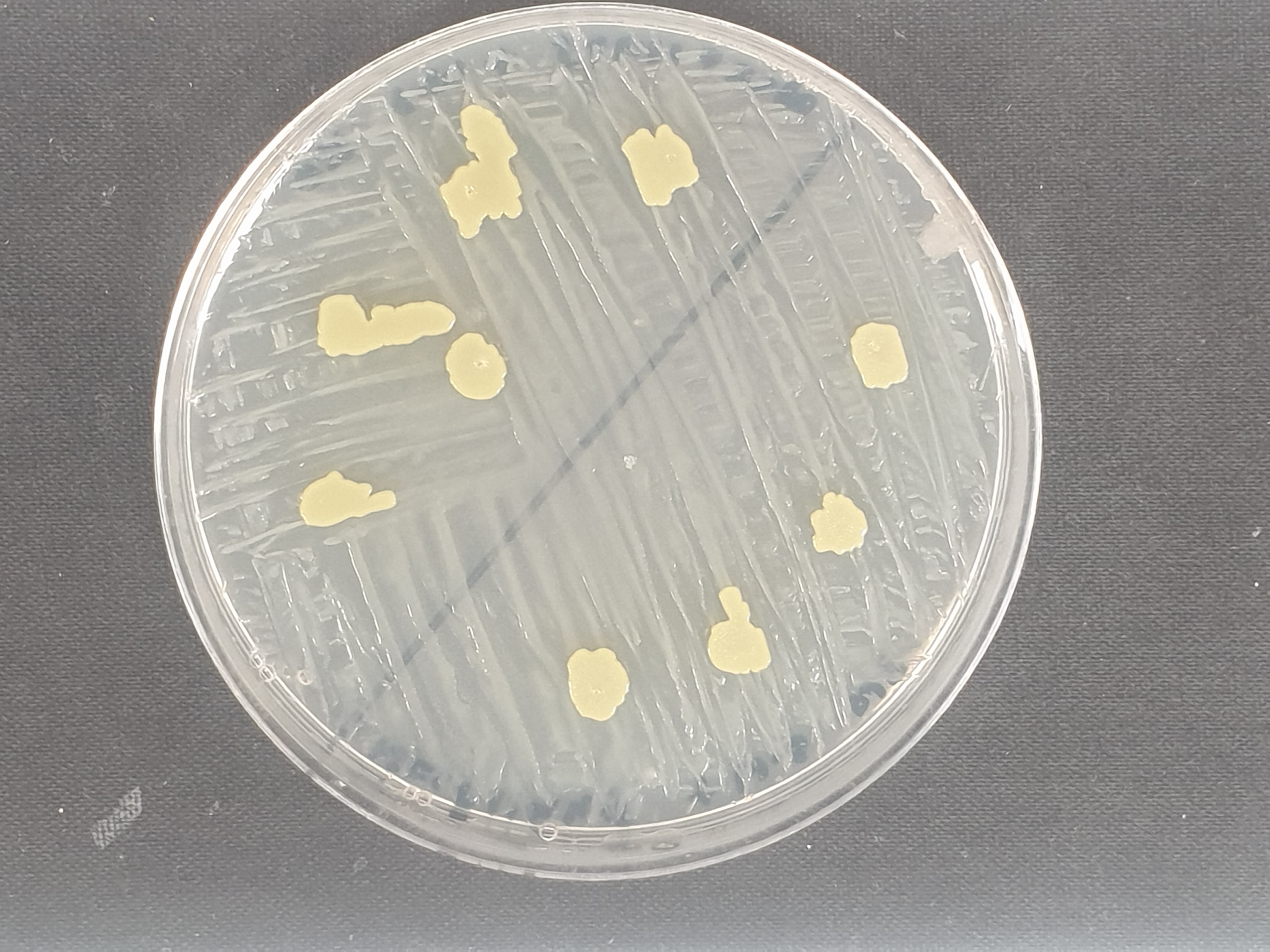

Supplement: Supplementary file 7 — (JPG 1.59 MB) [file 253_2026_13870_MOESM7_ESM.jpg]
