# Supplementary material for: Neisseria subflava Type 6 Secretion System competition with bacterial and fungal species
Source: Appl Microbiol Biotechnol. 2026 May 23;110(1):219. doi: 10.1007/s00253-026-13870-6 (PMC13375697; doi:10.1007/s00253-026-13870-6)
Supplement: Supplementary file 8 — (PDF 310 KB) [file 253_2026_13870_MOESM8_ESM.pdf]

The *Neisseria subflava* Type 6 Secretion System competition with bacterial and fungal species

Applied Microbiology and Biotechnology

Alan Calder, Lori A S Snyder (ORCiD 0000-0003-3172-3984)*

School of Life Sciences, Pharmacy, and Chemistry, Kingston University, Penrhyn Road, Kingston upon Thames, KT1 2EE, United Kingdom.

Corresponding author: L.Snyder@kingston.ac.uk

Supplementary

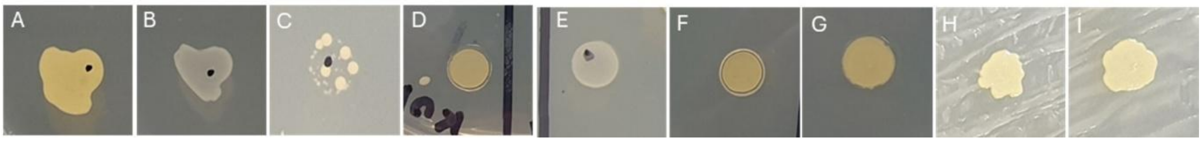

Supplementary Figure 1: An example of spots spreading out on the surface of GC agar for (A) *N. subflava* strain KU1003-01 and (B) *N. gonorrhoeae* strain NCCP11945. These were spotted without inoculation into a defined “rings” scored on the GC agar surface. (C) A competition spot consisting of *N. subflava* and *N. gonorrhoeae* added to GC agar from a mixture containing at low cell concentrations. (D) *N. subflava* strain KU1003-01 growing within a 5mm defined ring on GC agar. (E) *N. gonorrhoeae* strain NCCP11945 growing within a 5mm defined ring, scored on the surface of GC agar. (F) A competition spot consisting of the *N. subflava* KU1003-01 parent strain growing on top of *N. gonorrhoeae* strain NCCP11945. (G) A competition spot consisting of the *N. subflava* KU1003-01 knock-out strain growing on top of *N. gonorrhoeae* strain NCCP11945. (H) The *N. subflava* KU1003-01 parent strain growing on top of a lawn of *N. gonorrhoeae* strain NCCP11945. (I) The *N. subflava* KU1003-01 knock-out strain growing on top of a lawn of *N. gonorrhoeae* strain NCCP11945.

Supplementary Table 1. PCR primers used in generation of the *tssM* mutant.

**Primer name**	**Primer sequence**
HA1_KU1_*tssM*_fwd	5'- GATTGTATGTGCGAATAGC - 3'
HA1_KU1_*tssM*_rev	5'- CCGTTGAATATGGCTCATAGTAGAGTTTTCTCG - 3'
*kan*_fwd	5'- CGAGAAAACTCTACTATGAGCCATATTCAACGG - 3'
*kan*_rev	5'- CCATTGGGGTAATGTCATGATTAGAAAAACTCATCGAGC - 3'
HA2_KU1_fwd	5'- GCTCGATGAGTTTTTCTAATCATGACATTACCCCAATGG - 3'
HA2_KU1_rev	5'- GCAATCAATACCTGAAAGC - 3'
